# *A paradoxical switch*: the implications of excitatory GABAergic signaling in neurological disorders

**DOI:** 10.3389/fpsyt.2023.1296527

**Published:** 2024-01-10

**Authors:** Colin J. McArdle, Alana A. Arnone, Chelcie F. Heaney, Kimberly F. Raab-Graham

**Affiliations:** ^1^Department of Physiology and Pharmacology, Wake Forest University School of Medicine, Winston-Salem, NC, United States; ^2^Department of General Surgery, Wake Forest University School of Medicine, Winston-Salem, NC, United States

**Keywords:** GABA, depolarizing, excitatory, diseases, GABA_A_Rs, neurological

## Abstract

Gamma-aminobutyric acid (GABA) is the primary inhibitory neurotransmitter in the central nervous system. In the mature brain, inhibitory GABAergic signaling is critical in maintaining neuronal homeostasis and vital human behaviors such as cognition, emotion, and motivation. While classically known to inhibit neuronal function under physiological conditions, previous research indicates a paradoxical switch from inhibitory to excitatory GABAergic signaling that is implicated in several neurological disorders. Various mechanisms have been proposed to contribute to the excitatory switch such as chloride ion dyshomeostasis, alterations in inhibitory receptor expression, and modifications in GABAergic synaptic plasticity. Of note, the hypothesized mechanisms underlying excitatory GABAergic signaling are highlighted in a number of neurodevelopmental, substance use, stress, and neurodegenerative disorders. Herein, we present an updated review discussing the presence of excitatory GABAergic signaling in various neurological disorders, and their potential contributions towards disease pathology.

## Introduction

Gamma-aminobutyric acid (GABA), the primary inhibitory neurotransmitter in the adult brain, modulates the inhibitory-excitatory balance necessary for proper brain function predominantly through ionotropic GABA_A_ receptors (GABA_A_Rs) and metabotropic GABA_B_ receptors (GABA_B_Rs) (1, 2). Both GABA receptor subtypes play a critical role in neurotransmission and proper neuronal functioning. GABA_A_Rs are pentameric ligand-gated chloride (Cl^−^) channels located within the postsynaptic density (PSD) that rapidly hyperpolarize neurons (3, 4). Heterodimeric G-protein coupled receptors (GPCR) GABA_B_Rs are located extrasynaptically and generate prolonged and sustained inhibition by activating such channels like G-protein-coupled inwardly rectifying potassium (GIRK) channels and inhibiting voltage-gated calcium channels (VGCC) (1, 5).

**Figure 1:**
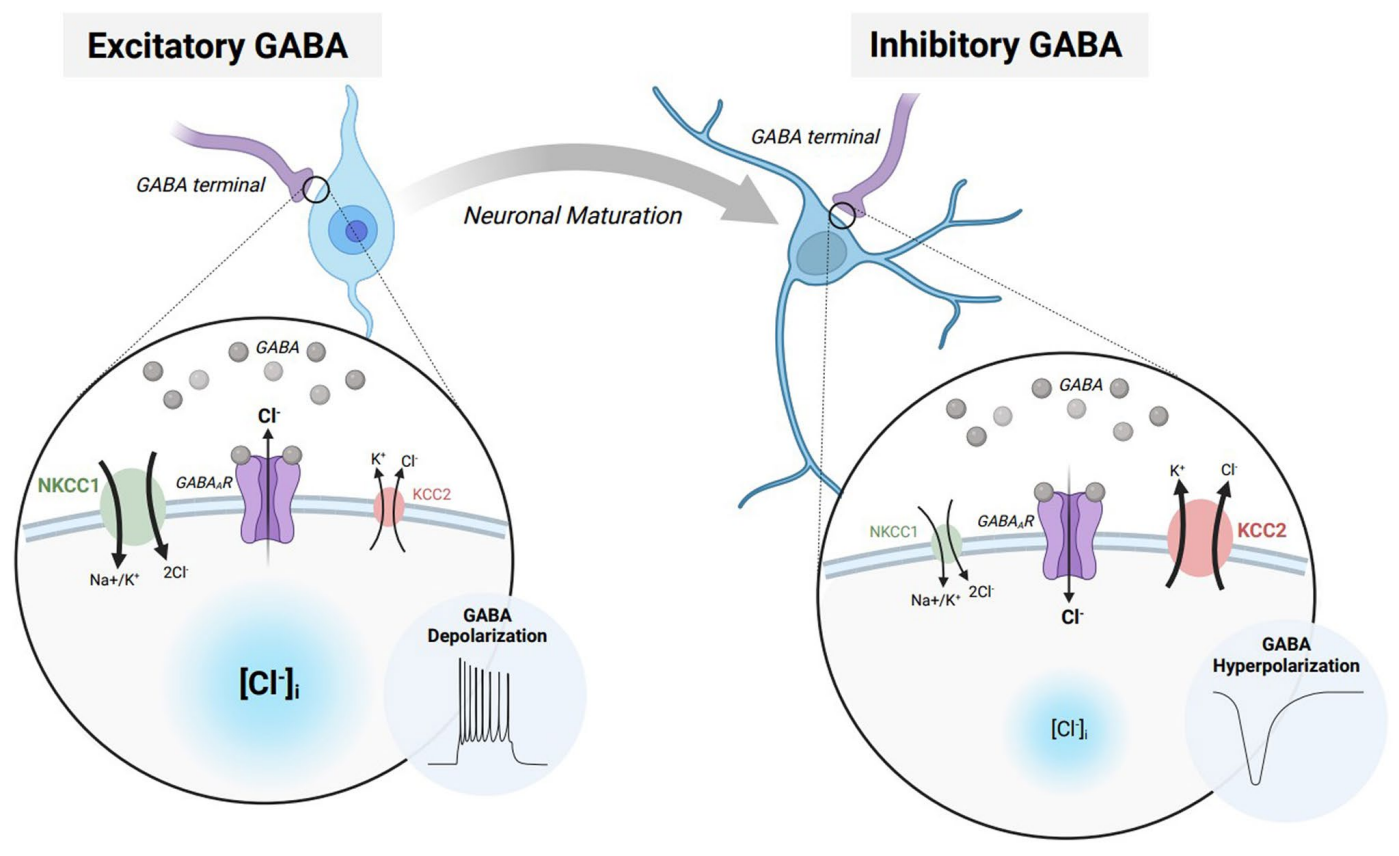
The excitatory/inhibitory transition of GABA during neurodevelopment and maturation. Immature neurons express elevated levels of Na-K-2Cl cotransporter 1 (NCKK1 – *green*) which allows for a greater intracellular chloride concentration while the neuron is at rest. When GABA binds to GABA_A_ receptors (GABA_A_Rs – *purple*), chloride flows down its respective concentration gradient, resulting in an efflux of chloride ions. The elevation in membrane potential furthermore causes the neuron to depolarize and elicit an action potential. Throughout neuronal maturation, NKCC1 expression is diminished while K-Cl cotransporter 2 (KCC2 – *red*) is elevated. Heighted KCC2 expression in mature neurons allow for a lower intracellular chloride concentration while the neuron is at rest. When GABA binds to GABA_A_Rs, the result is an influx of chloride ions. The decrease in membrane potential moreover causes the neuron to hyperpolarize and prevent action potential propagation. Figure created with BioRender.com.

The effects of GABA_A_R activation rely on the concentration gradient of permeable ions and the membrane potential of the cell, preventing an innate drive towards either excitation or inhibition (3). Specifically, the electrophysiological effects of GABA_A_R signaling highly rely on the intra/extracellular concentrations of chloride (Cl^−^) ions (Figure 1). During early development, GABAergic transmission is excitatory due to an increased concentration of intracellular Cl^−^ (6). Increased intracellular Cl^−^ results in an efflux of chloride upon GABA_A_R activation, raising the membrane potential to depolarize the neuron. This developmental profile of Cl^−^ is driven by a high expression of the Na^+^-K^+^-Cl^−^ 1 (NKCC1) cotransporter that maintains higher intracellular Cl-concentration during early development. Excitatory GABAergic transmission can additionally increase calcium concentration by activating N-methyl-D-aspartate receptors (NMDARs) (7), resulting in an increased probability for action potential firing (8). These GABA-mediated processes develop prior to the establishment of functional ionotropic glutamate receptors (9) and help to facilitate the maturation of the nervous system by promoting cell proliferation, migration, differentiation, and the expression of brain-derived neurotrophic factor (4, 6, 10, 11). Throughout later courses of development, the expression of the K^+^/Cl-cotransporter 2 (KCC2) maintains a lower intracellular Cl^−^ concentration. Lowered intracellular Cl^−^ results in an influx of chloride upon GABA_A_R activation, lowering the membrane potential to hyperpolarize the neuron (12, 13). The switch from excitatory to inhibitory GABAergic signaling results in a maturation of synaptic and network function in the brain, governing normal physiological behaviors into adulthood.

While excitatory GABAergic transmission is usually only observed during early development, specific populations of adult neurons continue to display excitatory responses to GABA_A_R signaling (14–16). However, pathological conditions can also influence the continued presence of GABA_A_R signaling in the mature brain (17). A number of mechanisms have been proposed that could underlie changes in GABA activity, including brain-region specific alterations in GABA_A_Rs, changes in Cl^−^ homeostasis, or plasticity of GABAergic synapses. The mechanisms responsible for disrupting the switch from excitatory to inhibitory GABAergic signaling additionally have the potential to exacerbate the various molecular and behavioral pathologies observed in neurological disorders. Herein, this review will highlight the current literature discussing the presence of paradoxical, excitatory GABAergic signaling in the mature nervous system and their contributions towards the hallmarks of several neurological disorders.

## Stress related disorders

Stress, either acute or chronic, correlates with an increased prevalence of several behavioral phenotypes including anxiety, depression, learning impairments, seizure susceptibility, and substance use (18). The physiological response to stress is regulated through the Hypothalamus-Pituitary–Adrenal (HPA) Axis where the release of corticotropin-releasing hormone (CRH) from the paraventricular nucleus (PVN) of the hypothalamus stimulates the release of adrenocorticotropic hormone (ACTH) from the pituitary gland to further release cortisol from the adrenal glands (18). Several brain regions, neurotransmitters, and hormones influence the HPA-axis activity, however, GABAergic projecting neurons that synapse onto neurons in the PVN are the primary regulator of the physiological stress response (19).

The addition of one or multiple stressors results in persistent activation of the HPA-axis and subsequent negative behaviors (20–22). The downstream hyperactivation of this axis is furthermore hypothesized to result from alterations in GABAergic signaling. In preclinical models, acute and chronic stress has been shown to alter the shift from excitatory to inhibitory GABAergic signaling in the brain. Previous research utilizing several stress paradigms in rodents including restraint stress, maternal separation, hyperosmotic stress, prenatal stress, early-life stress, or a combination of different stressors all denote a depolarizing shift in GABA_A_R-mediated signaling in the hypothalamus (23–32). Herein, will focus on stress induced mechanisms that shift GABA_A_R signaling from hyperpolarizing to depolarizing.

### Stress and neurosteroids

The stress-induced switch in GABA_A_R function is hypothesized to be mediated through several mechanisms: interactions from neurosteroids, altered GABA synthesis/release, as well as chloride (Cl^−^) dyshomeostasis. Neurosteroids, or steroids synthesized in the brain, adrenals, and gonads, modulate neuronal excitability by allosterically binding to GABA_A_Rs (33). Previous work has shown that neurosteroidogenesis, or the synthesis of neurosteroids, exacerbates the excitatory GABAergic drive in response to stress (32). Acute stress can elevate neurosteroids, which can bind and modulate GABA_A_R function (17). In a model of acute restraint stress, GABA depolarizes CRH-positive neurons in the PVN and further increases corticosterone levels. Acute stress additionally elevates the neurosteroid, tetrahydrodeoxycorticosterone (THDOC), which exacerbates excitability of CRH-positive neurons. Furthermore, inhibiting neuosteroidogenesis with finasteride reduces stress-induced anxiety-like behaviors in mice.

### Stress and transsynaptic GABA signaling

**Figure 2:**
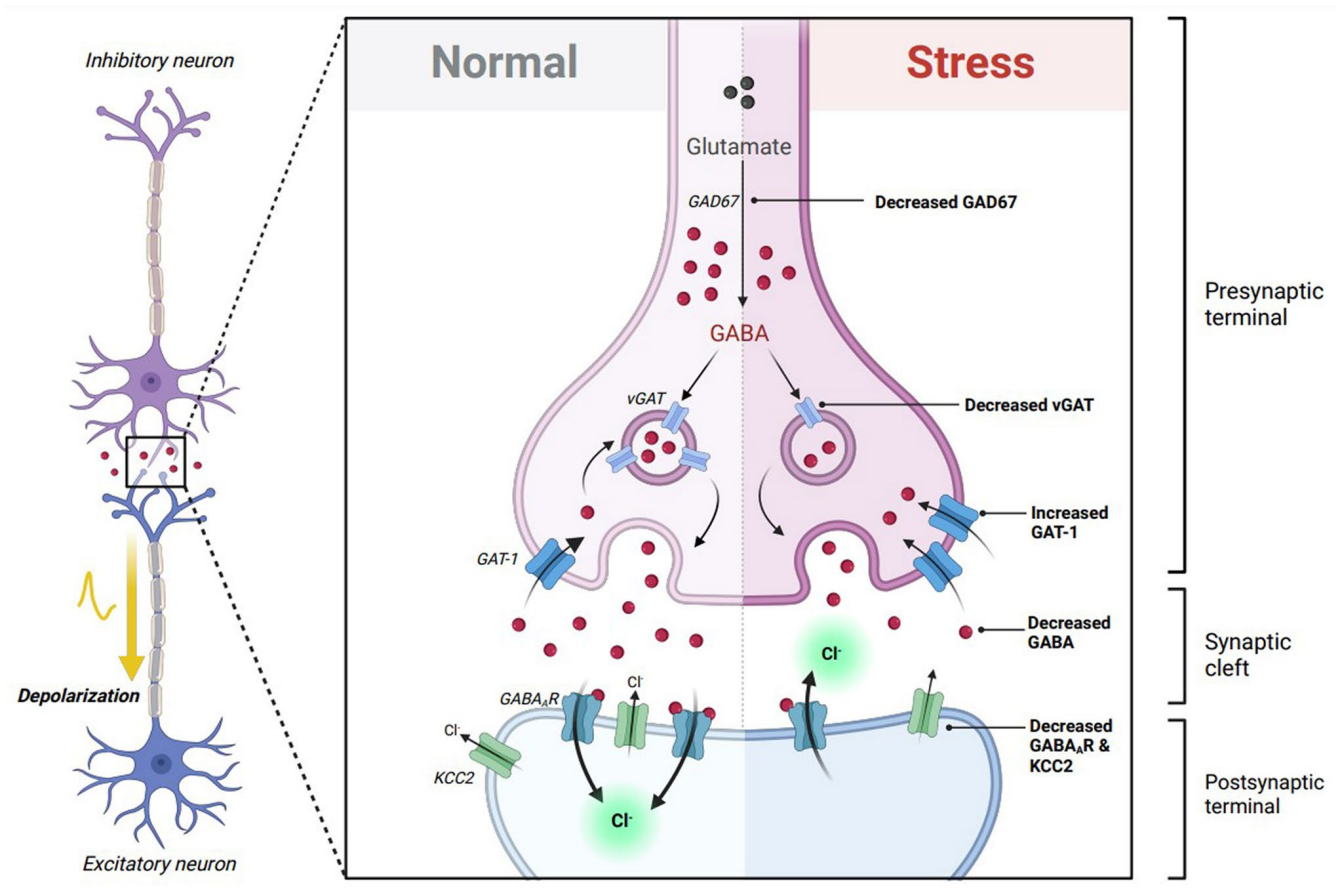
Theoretical effects of GABA transsynaptic signaling on excitatory GABAergic responses. GABA itself is critical in transitioning GABA_A_ receptor (GABA_A_R) responses from depolarizing to hyperpolarizing throughout neuronal maturation. In immature neurons, GABA binding to excitatory GABA_A_Rs promotes the expression of K-Cl cotransporter 2 (KCC2), which decreases intracellular chloride concentration, allowing future GABA_A_R-mediated responses to become inhibitory in mature neurons. Stress exposure diminishes GABA transsynaptic signaling by reducing glutamic acid decarboxylase 67 (GAD67) and vesicular GABA transporter (vGAT) expression, while elevating GABA transporter type 1 (GAT-1) expression. This results in lowered extracellular GABA to freely bind to its respective receptors. Stress also reduces the expression of GABA_A_Rs via downregulation of several GABA_A_R subunits. The lack of available GABA to bind to GABA_A_Rs following stress hypothetically reduces KCC2 expression, which disturbs chloride homeostasis and promotes an excitatory switch in GABAergic signaling. Figure adapted from “Mechanism of action of Selective Serotonin Reuptake Inhibitors,” by BioRender.com (2023). Retrieved from https://app.biorender.com/biorender-templates.

Excitatory GABA_A_R-mediated responses following stress can also be mediated through changes in transsynaptic GABA signaling. This can include alterations in GABA synthesis/transport, as well as changes in GABA_A_R protein expression. Previous studies denote a reduction in endogenous GABA throughout the nervous system following stress (17, 34, 35). It is also important to note that GABA itself acts as a limiting factor to promote the transition from depolarizing to hyperpolarizing GABA_A_R-mediated responses by upregulating KCC2 mRNA expression (6). Changes in stress-induced GABA levels can furthermore be attributed to its synthesis, and transport. Glutamic acid decarboxylase 67 and 65 (GAD67 and GAD65), which synthesize GABA from glutamic acid, is downregulated during periods of stress (34, 36, 37). Vesicular GABA transport (vGAT), which imports presynaptic GABA into vehicles, is also reduced following chronic stress (38, 39). Finally, gamma-aminobutyric acid transporter-1 (GAT-1), which is responsible for the reuptake of GABA, is increased after stress (36). Furthermore, these molecular alterations can result in a net decrease in GABA levels, potentiating the presence of stress-induced depolarizing GABA_A_R-mediated responses (Figure 2).

Different stress paradigms alter GABA_A_R expression in different areas of the brain (17). Various stress paradigms in preclinical models exhibit a reduction in GABA_A_R subunit expression (39–43), as well as hyposensitivity of these receptors towards GABA binding (34, 35). To note, postsynaptic GABA_A_Rs and KCC2 co-transporters are shown to display a bidirectional crosstalk that influences their function. As both colocalize along dendrites (44), GABA_A_R activity promotes the surface stability of KCC2 via the chloride-sensing kinase, WNK1 (45). Conversely, elevated KCC2 activity potentiates the expression and function of hyperpolarizing GABA_A_Rs (46–48). Furthermore, it can be hypothesized that stress-induced reductions in GABA_A_R expression can lead to reduced KCC2 stability and chloride dyshomeostasis, allowing for the presence of excitatory GABAergic signaling.

### Stress and chloride homeostasis

Chloride (Cl^−^) homeostasis, which is essential for maintaining effective GABAergic inhibition, is also affected by stress. Several reports have indicated that stress-induced Cl- dyshomeostasis is linked to either decreases in KCC2 or increases in NKCC1 within the hypothalamus (24, 25, 27, 28, 49, 50). As previously discussed, downregulation of the KCC2 cotransporter results in elevated intracellular chloride concentrations, allowing GABA_A_R-mediated responses to switch from hyperpolarizing to depolarizing. Additional studies also note dephosphorylation of the KCC2 residue Ser940 following stress (17, 30–32). The phosphorylation of Ser940 is dependent on Protein kinase C (PKC) activity and enhances KCC2 surface stability and activity by reducing endocytosis (51). Furthermore, reduced phosphorylation and surface expression of KCC2 provides a molecular mechanism underlying Cl- dyshomeostasis and excitatory GABAergic signaling following stress.

One study, however, indicates that stress accelerates the excitatory GABA switch in the infralimbic cortex at postnatal day (PND) 9 (52). Additionally, NKCC1 mRNA expression was downregulated at an earlier developmental timepoint following stress. An explanation for the differences between this study and those previously discussed is that the neurological effects of stress may not be uniform across each brain region. Stress has been shown to reduce PFC function in both humans and animal models that furthermore impairs learning and memory (53). These changes in the PFC are additionally correlated with alterations in network connectivity and dendritic remodeling. Interestingly, the PFC has previously been shown to inhibit HPA-axis activity (54). An acceleration towards hyperpolarizing GABAergic signaling and NKCC1 downregulation moreover could contribute to reductions in PFC activity following stress. These molecular alterations in the PFC could therefore exacerbate HPA-axis activity in response to stress. Additionally, the stress paradigm used in this study did not show elevations in cortisol, which is an output measure for HPA-axis activity. Therefore, the effects from this paradigm may not classically influence HPA-axis activity as compared to the previously discussed research, resulting in a differential physiological and molecular response to stress.

### ‘Anti-stress’ mechanisms and KCC2 stability

*M*echanisms altering GABAergic signaling are involved in both promoting and counteracting the negative behavioral effects of stress. Oxytocin, a hormone released from the pituitary gland, has been previously shown to have therapeutic properties by initiating anxiolytic responses following stress (55, 56). These ‘regenerative’ properties of oxytocin promote learning and memory, cognition, and social behaviors (55, 57, 58). Oxytocin counterbalances the stress response by modulating the GABA switch from excitatory to inhibitory. As previously discussed, stress paradigms promote depolarizing GABA_A_R-mediated responses by downregulating KCC2 expression. On the contrary, oxytocin initiates the switch from excitatory to inhibitory GABAergic signaling by regulating KCC2 activity. In the hippocampus, oxytocin was previously shown to increase the phosphorylation of KCC2 via the Oxtr/G_q_/PKC pathway, increasing KCC2 surface stability to shift GABAergic signaling towards inhibitory during early stages of development (59). Hippocampal neurons lacking oxytocin receptors (Oxtr^−/−^) exhibit a delay in switching from excitatory to inhibitory GABAergic responses, as well as a net elevation in neuronal excitability (59). During childbirth, a surge in maternal levels of oxytocin allows the fetal brain to transition from depolarizing to hyperpolarizing GABA_A_R-mediated signaling (60), promoting healthy development and maturation of the brain during early development. In human populations, diminished oxytocin has been shown in patients with anxiety and post-traumatic stress disorder (PTSD) (61–63). The molecular mechanisms underlying oxytocin’s effects on restoring inhibitory GABAergic signaling can furthermore validate oxytocin’s therapeutic potential in alleviating negative behaviors and symptoms in patients with stress related disorders.

## Alzheimer’s disease and dementia

The most common form of dementia, Alzheimer’s disease (AD), is marked by memory impairment and cognitive decline (64). These behavioral abnormalities are simultaneously paired with molecular hallmarks including amyloid-beta (Aβ) plaque and neurofibrillary tangles. In addition to these major hallmarks, patients and preclinical models display profound neuronal hyperexcitability and glutamate excitatory that is aversive towards neuronal and synaptic function (65–68). The majority of research hypothesizes overactive NMDAR receptors signaling as the primary contributor towards hyperexcitability in AD (69, 70), however, several studies indicate excitatory GABAergic signaling as an additional contender towards hyperexcitability and the pathology present during the AD disease progression.

Switches in GABAergic signaling have previously been characterized in a preclinical rodent model of AD-related pathology. 2 and 3-month-old rats injected with Aβ_1-40_ fibrils exhibit a reduction in hippocampal IPSC amplitude compared to wildtypes (71). Of note, Aβ_1-40_ treatment also results in a positive shift in the equilibrium potential of chloride ions (E_Cl-_), indicating that Aβ_1-40_ fibrils promote an excitatory GABAergic shift in the hippocampus. This study additionally found similar electrophysiological trends in a 9-month-old APP/PS1 transgenic model of AD. APP/PS1 hippocampal slices also show reduced IPSC amplitude and a positive E_Cl-_ shift, emphasizing the conservation of excitatory GABAergic signaling across multiple preclinical AD models.

Previous studies have also uncovered the presence of excitatory GABA switches in additional models of AD-related pathologies. A transgenic mouse line expressing recombinant neutralizing anti-nerve growth factor (NGF) antibodies (AD11 mice) display similar pathological hallmarks to AD such as amyloid plaque and neurofibrillary tangle deposition, neurodegeneration, long-term potentiation (LTP) deficits, and behavioral abnormalities (72–75). 6-month-old AD11 hippocampal slices with isoguvacine, a GABA_A_R agonist, increased action potential frequency in CA1 hippocampal neurons (76). The increase in firing was further abolished using bicuculine or gabazine, two GABA_A_R antagonists. To determine the presence of an excitatory switch in GABAergic signaling, hippocampal GABA_A_R-mediated postsynaptic currents (EGPSC) were recorded via perforated patch clamping following stimulation of local GABAergic interneurons. In wild-type mice, EGPSCs are reduced in comparison to the respective resting membrane potential. In AD11 mice, however, EGPSCs are increased with respect to the resting membrane potential. The positive shift in GABA_A_R-mediated current in AD11 mice indicates an aberrant shift in hyperpolarizing GABAergic activity to depolarizing.

In conjunction to the electrophysiological findings that GABA acts to depolarize neurons in several models of AD-related pathology, previous research has investigated molecular changes that could underlie the GABA switch. As noted before, a developmental switch from NKCC1 to KCC2 expression underlies the excitatory to inhibitory shift in GABAergic signaling under normal conditions. However, data from both preclinical models and patients with AD show that this transition is disturbed. Imbalances between NKCC1 and KCC2 have recently been shown in patients with AD (77, 78) that are exacerbated with the onset of epileptic activity, indicating that hyperexcitability can act as a feed-forward mechanism to promote excitatory GABAergic signaling. Previous findings in preclinical models of amyloid-beta deposition (APP/PS1 transgenic mice and Aβ_1-40_ fibril treatment) note reduced KCC2 protein expression in the hippocampus and cortex (71, 76, 79, 80) with increases in NKCC1 protein expression (81). One study, however, indicates increases in KCC2 in a different model of AD-related pathology. Discrepancies between results can be due to contrasting animal models between these studies. KCC2 expression is shown to increase with treatment of AβO oligomers while it decreases with Aβ_1–40_ fibril treatment. Aβ oligomers and fibrils display different chemical structures and have been shown to have differential effects on neuronal function throughout the progression of Aβ plaque deposition (82). KCC2 expression could alter based on the current changes in neuronal activity induced by either Aβ oligomers or fibrils. Therefore, further exploration is required to definitively confirm the effects of Aβ plaque and different structural forms of Aβ on chloride homeostasis and excitatory GABAergic signaling. Interestingly, treatment with γ-secretase inhibitor, LY411575, restored KCC2 levels in APP/PS1 mice, highlighting the direct impact of Aβ plaque on KCC2 expression (80). Finally, in an APOE4-KI mouse, a model for the largest genetic risk factor of AD, also presents with an increased ratio between NKCC1 and KCC2 expression (83). Moreover, the consensus amongst the current literature argues a predominance towards NKCC1 expression that can underlie the depolarizing actions of GABA in models of AD-related pathology.

It is important to note that while studies have explored the relationship between AD-related pathology and excitatory GABAergic switches, previous research has explored the link between non-pathogenic amyloid precursor protein (APP) and alterations in GABAergic signaling. APP is the precursor protein to Aβ plaque and is metabolized through several steps to produce its subsequent products. The presence of either α-secretase or β-secretase can influence APP’s processing into non-pathogenic or pathogenic products, respectively. While APP itself is not considered toxic in the brain, studies have shown contradicting results in APP’s role in GABAergic transmission. In an APP^−/−^ model, CA1 hippocampal slices display a positive shift of the GABA reversal potential (E_GABA_), reduced IPSC amplitude, and reduced GABA_A_Rα1 expression. The findings additionally show that APP^−/−^ mice exhibit reduced KCC2 surface expression. This is further linked to APP’s ability to physically interact and stabilize KCC2 to the plasma membrane to prevent its ubiquitination and degradation (82). In opposition, *in vitro* cortical neurons transfected to express full-length neuronal wildtype human APP increase [Ca^2+^]_i_ and action potential frequency in response to treatment with GABA. Of note, overexpression of hAPP in cortical neurons resulted in decreased KCC2 expression with no differences in NKCC1 in both *in vitro* and *in vivo* models (78). Differences in these results may be due to the age in which KCC2 expression was examined in mice. The first study (82) utilized an older APP^−/−^ mouse model while the second study (78) utilized younger hAPP transfected cortical cells and mouse models. Neurons were transduced to overexpress APP prior to when GABA is predicted to shift from depolarizing to hyperpolarizing, while measurements in the APP^−/−^ model were done after the switch. Furthermore, it can be hypothesized that APP has differential effects on KCC2 expression and neuronal hyperexcitability pre- and post-GABA switch.

**Figure 3:**
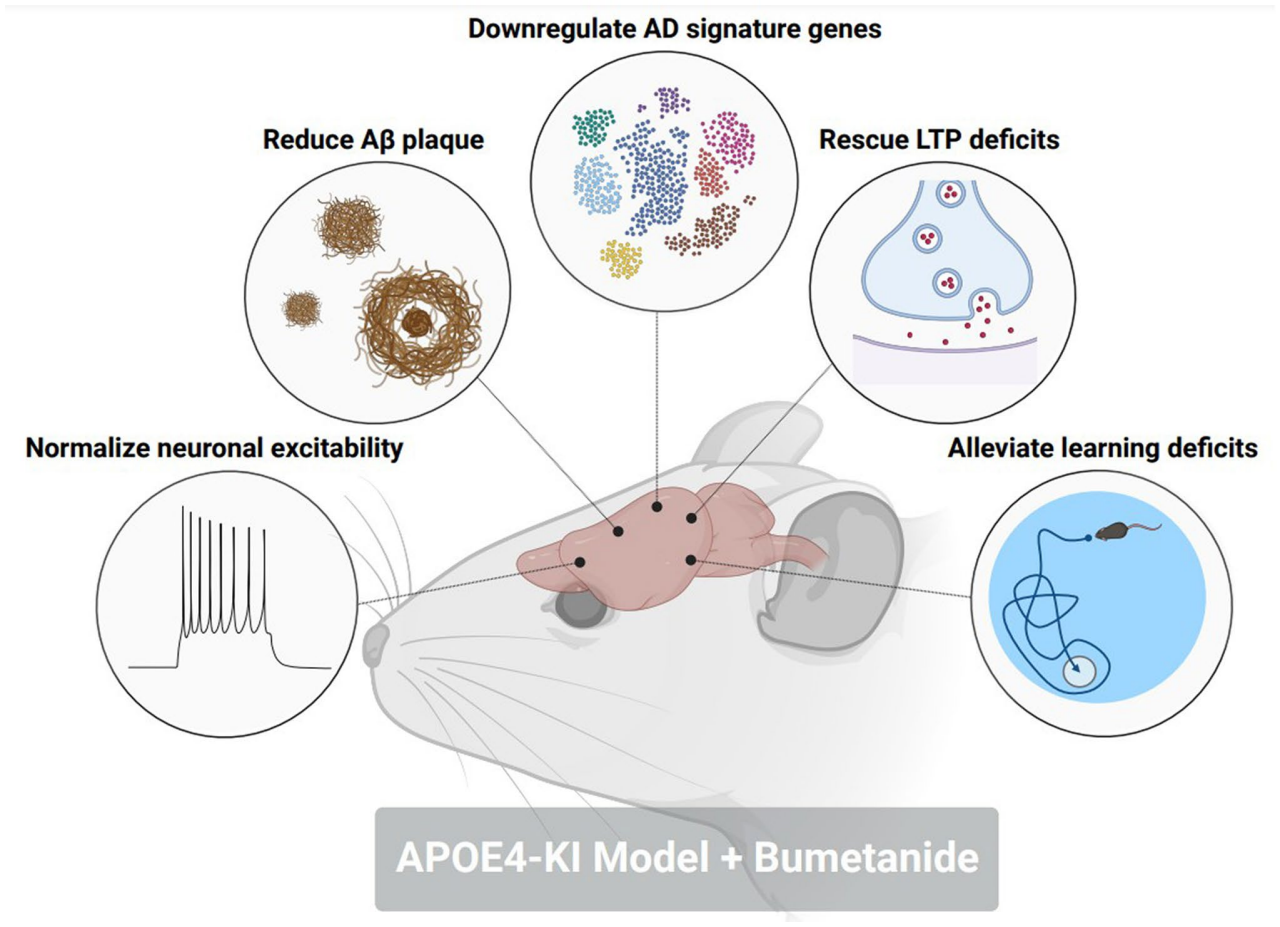
Bumetanide alleviates pathological, electrophysiological, and behavioral hallmarks in an AD-related pathology mouse model. Treatment with NKCC1 inhibitor, bumetanide, in an aged APOE4-KI mouse corrected the following pathological phenotypes in a model of AD-related pathology: (1) normalized neuronal excitability in CA1 pyramidal neurons. (2) reduced amyloid-beta plaque size and number in the hippocampus and cortex. (3) downregulated the expression of AD signature genes that promote disease pathology. (4) rescued deficits in long-term potentiation (LTP) within the CA1 of the hippocampus. (5) Alleviated deficits in spatial memory through a Morris water maze. Figure created with BioRender.com.

To further probe the contributions of excitatory GABAergic signaling towards memory and cognitive dysfunction in AD, several studies have investigated the impacts of restoring GABAergic transmission as a potential therapeutic target in preclinical and clinical settings. In rodent models of AD-related pathology, treatment with bumetanide, an NKCC1 inhibitor, significantly enhances contextual fear learning and spatial learning (71, 81). Interestingly, a study (84) identified bumetanide as a top ranking therapeutic for APOE4-related AD according to a Connectivity Map (CMap) database. Bumetanide treatment in an APOE4-KI mouse model mitigated neuronal hyperexcitability, restored LTP deficits, decreased Aβ plaque deposition, and rescued spatial learning deficits (Figure 3). APOE4-KI mice treated with bumetanide also attenuated transcriptomic deficits in genes relating to GABAergic synaptic function and circadian rhythms, two biological processes that are affected by AD-related pathology. This study furthermore highlighted that exposure to bumetanide in humans was correlated with lowered AD prevalence according to electronic health record databases. Of note, a previous study (85) showed that a 7-day regimen of daily injections of 25 mg/kg Memantine, an NMDA receptor antagonist that is a commonly prescribed to patients with AD, reduced cortical and hippocampal KCC2 expression while simultaneously, attenuated the behavioral response to GABA_A_R activation with diazepam. This data brings forth the use of alternative FDA-approved medications, such as bumetanide, that can restore homeostatic GABAergic signaling as a potential therapeutic avenue to prevent memory and cognitive impairments in AD patients.

Although previous research investigating excitatory GABAergic signaling in AD has predominately focused on models of Aβ deposition, there is a lack of evidence stating whether an excitatory GABA switch is present in models of hyperphosphorylated tau and neurofibrillary tangles. Currently, there are sufficient data to argue for a reduced inhibitory GABAergic tone in preclinical models of tau tangles (86, 87); however, no findings have been presented as to whether a shift to excitatory GABAergic neurotransmission contributes to diminished inhibitory neuronal signaling.

## Autism spectrum disorders

### Autism spectrum disorder – clinical studies

Autism Spectrum Disorder (ASD) is a neurodevelopmental disorder commonly presented with symptoms such as repetitive behaviors and impairments in social communication (88). Due to the heterogeneity of this disorder, patients display a spectrum of symptoms that is accompanied by a specialized regimen of combinational therapies and pharmaceutical treatments. Previous research has interestingly noted a presence of excitatory GABAergic signaling that can potentially underlie behavioral symptoms (89). Children with ASD were previously treated with diazepam, a GABA_A_R positive allosteric modulator, that typically exerts an anxiolytic effect. On the contrary, diazepam paradoxically increased aggressive behavior in ASD patients (89), indicating a dysfunction in GABAergic signaling.

KCC2 variants have furthermore been identified in human populations with ASD (90). Genomics and exosome sequencing identified a *SLC12A5* variant, the gene encoding for KCC2, that is prevalent in patients with ASD. These variants include R952H, R1O49C, and R1048W within the C-terminal regulatory domain of KCC2, compromising the ability of KCC2 to maintain low concentrations of intracellular chloride and hyperpolarizing GABAergic responses. These KCC2 variants are also predicted to target the CpG sites on the *SLC12A5* gene, which can affect the likelihood of DNA methylation and repressed gene expression. Moreover, this data highlights a distinct molecular mechanism in which depolarizing GABAergic responses are preserved in ASD patients. An overabundance of non-functional KCC2 variants can result in disturbed chloride homeostasis, promoting an excitatory GABAergic drive. Of note, several clinical studies have utilized bumetanide as a novel therapeutic for patients with ASD (91–93). Collectively, the evidence shows promising effects of bumetanide to alleviate behavioral and electrophysiological abnormalities. These studies furthermore highlight the significant contributions of excitatory GABAergic towards the pathogenesis of ASD.

### Fragile X syndrome

Fragile X Syndrome (FXS) is the most common genetic form of intellectual disability and autism spectrum disorder (ASD). FXS is caused by an overabundance of CGG motif repeats on the *Fmr1* gene, silencing its corresponding protein product, Fragile X Ribonuclear Messenger Protein 1 (FMRP) (94). FMRP is an RNA-binding protein that regulates a large domain of proteins critical for transsynaptic signaling (95). The loss of FMRP furthermore results in synaptic dysfunction as well as imbalances in excitatory/inhibitory transmission. This imbalance could furthermore be linked to excitatory GABAergic signaling. Pyramidal neurons in layers IV and V of the somatosensory cortex from WT mice display a shift from depolarizing to hyperpolarizing GABAergic signaling at approximately postnatal day (PND) 9 (96). Neurons from *Fmr1* KO mice, however, display a delay in this shift, switching to hyperpolarizing GABAergic responses at PND 14 (96). Additional reports have stated similar delays in hyperpolarizing GABAergic signaling or disrupted responses to GABA (96–99). One study does report a hyperpolarizing GABA switch to occur earlier in *Fmr1* KO slices (~P21–25) in auditory cortical neurons as compared to wildtypes (100). However, the region of interest differs from the previous literature investigating the somatosensory cortex. Disturbances to shift towards hyperpolarizing GABA can potentially differ across cortical subregions, explaining the discrepancy of exactly when GABA is no longer excitatory throughout the FXS disease progression.

Depolarizing GABA response in FXS is further hypothesized to be contributed from chloride ion imbalances, via upregulated NKCC1 expression (96), with either decreases or no changes in KCC2 expression (96, 99) in *Fmr1* KO mice. FMRP is known to repress the majority of its target synaptic mRNAs (95). Two of its targets include *SLC12A5* and *SLC12A2* mRNAs, which encode for KCC2 and NKCC1, respectively. However, no conclusive evidence states whether FMRP in fact represses the synthesis of KCC2 and NKCC1. Silencing of FMRP in FXS could furthermore result in an overabundance of either KCC2 or NKCC1, leading to disturbances in chloride homeostasis and alterations in excitatory GABAergic signaling. Previous work also denotes reduced membrane expression of KCC2 in *Fmr1* KO cortical tissue. Further analysis of the *Fmr1* KO cortices indicate an absence of several phosphorylation sites within the intracellular C-terminus of KCC2 (101). Reduction in KCC2 phosphorylation in FXS could result in compromised membrane trafficking or surface stability.

Studies have additionally targeted NKCC1 in FXS to alleviate symptoms in preclinical models. Treatment with bumetanide, an NKCC1 inhibitor, during a critical period of development (P0-P10) restored hyperpolarizing GABAergic responses in the somatosensory cortex of *Fmr1* KO mice, normalized neuronal hyperexcitability, and rescued synaptic function amongst thalamocortical excitatory synapses (102). Bumetanide also altered the proteome in the somatosensory cortex in FMR1 KO mice to overexpress proteins relating to inhibitory interneuron development while downregulating at-risk proteins of several developmental disorders (102). Interestingly, an additional study corroborated these findings by pretreating pregnant FXS female mice with bumetanide and monitoring electrophysiological behavioral phenotypes of their offspring. Maternal bumetanide pretreatment restores hyperpolarizing GABA responses and reduces excitability in the hippocampus of offspring (99). Ultrasonic vocalization behaviors and brain oscillations are also normalized in pretreated offspring (99).

### Rett syndrome

Rett Syndrome is a developmental disorder predominately characterized by speech dysfunctions, gait abnormalities, motor impairment, and disturbed breathing patterns (103). The disease is caused by mutations in the Methyl-CpG-binding protein 2 (MeCP2) which under physiological conditions, is critical in regulating gene expression in the brain (103). Previous research denotes an imbalance between excitatory and inhibitory transmission that can underlie the behavioral symptoms in Rett patients. In both *in vitro* and *in vivo* models of Rett syndrome where MeCP2 is absent (Mecp2^y/−^), neurons exhibit a depolarizing response to GABA (104–106). Studies have additionally found a similar pattern compared to other ASD-related diseases such that KCC2 mRNA and protein expression is reduced in preclinical models and Rett patients (104, 106–108). One study identified a potential mechanism underlying reduced KCC2 expression. REST, a transcriptional repressor competes with MeCP2 for the RE-1 binding site on the *SLC12A5* gene (109). In Rett neurons, where there is an absence of MeCP2, REST can occupy the RE-1 binding site, preventing the transcription of KCC2 (*SLC12A5*) mRNA (106).

Several studies have utilized different therapeutic approaches to alleviate excitatory GABAergic signaling and pathological symptoms in preclinical models of Rett Syndrome. Similar to studies done in FXS mice (99), maternal pretreatment with bumetanide 1 day prior to delivery restored the GABA switch, normalized excitability, increased hypersynchronization, and rescued impaired mGluR-induced LTD in CA3 pyramidal neurons of Mecp2^−/y^ mice (105). Another previous study developed a high-throughput drug screening platform to identify pharmaceuticals that could enhance KCC2 expression. A subset of small molecules coined KCC2 expression-enhancing compounds (KEECs) were selected which have a range of molecular targets including fms-like tyrosine kinase 3 (FLT3), glycogen synthase kinase 3 (GSK3), sirtuin 1 (SIRT1), and transient receptor potential cation channel subfamily V member 1 (TRPV1). These molecules were successful in elevating KCC2 expression. Treatment with KEECs not only restored hyperpolarizing GABAergic responses and excitability in MeCP2 KO cultured neurons, but also prevented morphological deficits in these neurons as well. Further treatment of KEECs in MeCP2-mutant mice also prevented the onset of locomotor and breathing abnormalities (110). Interestingly, several studies have utilized Insulin-like Growth Factor 1 (IGF-1) as a novel therapeutic alternative. Previous reports show IGF-1 treatment prevents behavioral, synaptic, and electrophysiological abnormalities in MeCP2-mutant mice (111, 112). Further evidence shows that IGF-1 mitigates its therapeutics effects in MeCP2-mutanat mice by elevated KCC2 and downregulated NKCC1 expression, therefore, lowering intracellular chloride levels needed to induce a hyperpolarizing GABAergic response. IGF-1 has previously been reported to accelerate the maturation of neuronal networks via KCC2 upregulation (113), granting this as a potential therapeutic avenue to alleviate developmental delays in Rett Syndrome.

### Tuberous sclerosis complex

Tuberous Sclerosis Complex (TSC) is an autosomal dominant disorder that results in the loss of either the *Tsc1* or *Tsc2* gene. Patients with TSC present with several autistic-like behaviors, but more importantly, experience at least one epileptic event in their lifetime (114, 115). Studies in clinical and preclinical models point to an imbalance between excitation and inhibition that favors a shift towards cortical hyperexcitability as the hypothesized cause of seizure susceptibility (114, 116, 117). Studies note alterations in GABAergic responses can contribute to aberrant excitability in TSC. In both TSC human cortical neurons and Xenopus oocytes that express membranes from TSC cortical tubers display either depolarizing GABA_A_ receptor-mediated responses or a significant delay in the excitatory GABA switch (118, 119). As a common mechanism between the previously discussed autism spectrum disorders, KCC2 expression is downregulated while NKCC1 expression is elevated in TSC neurons (118, 119). Of note, immunohistochemical evidence shows an elevated abundance of NKCC1 expression amongst dysplastic neurons in TSC corticies, while KCC2 expression is lowered within the soma and proximal dendrites (119). Several studies have utilized bumetanide to target NKCC1 function in TSC patients and noticed an attenuation in both behavioral and electrophysiological abnormalities with treatment (120–122).

**Figure 4:**
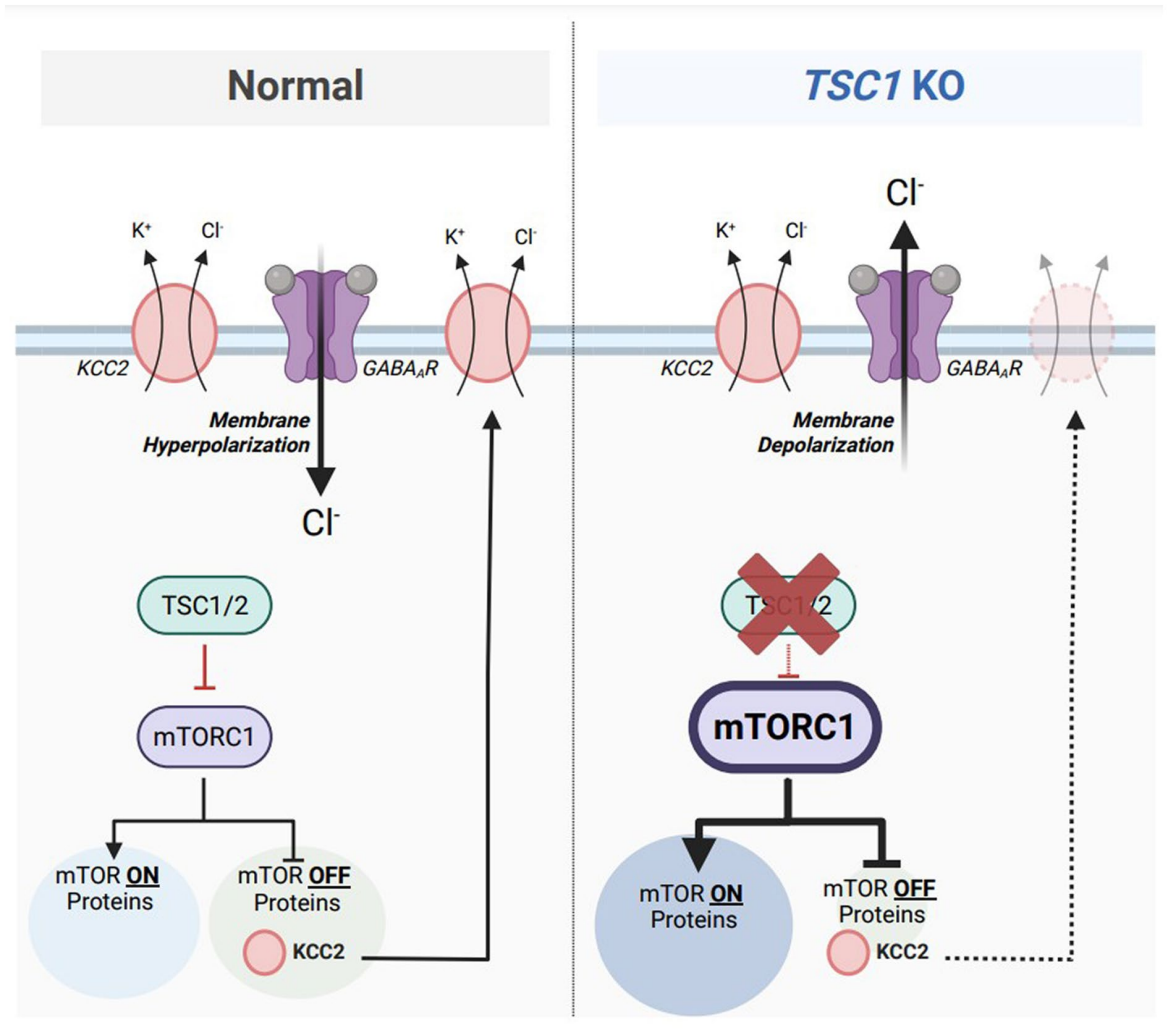
Aberrant mTORC1 activity represses KCC2 expression in a model of Tuberous Sclerosis Complex (TSC). Under normal conditions **(left)**, mammalian target of rapamycin complex 1 (mTORC1) equally activates the translation of 155 target mRNAs (mTOR ON proteins) and represses the translations of 166 target mRNAs (mTOR OFF proteins). Tuberous sclerosis proteins 1 and 2 (TSC1/2) acts upstream of mTORC1 and represses its activity. One particular mTOR OFF protein is KCC2, which is synthesized when mTORC1 is inactive. KCC2 is responsible for maintaining a low intracellular chloride concentration to maintain inhibitory GABA_A_R-mediated responses. In TSC1 KO neurons, a model of Tuberous Sclerosis Complex (TSC) **(right)**, TSC1/2 are absent which results in aberrant mTORC1 activity. The population of mTOR ON proteins are upregulated while the population of mTOR-OFF proteins are downregulated. Furthermore, KCC2 downregulation from elevated mTORC1 activity results in an accumulation of intracellular chloride, resulting in depolarizing GABA_A_R-mediated responses. Figure created with Biorender.com.

TSC is considered an mTORopathy, meaning that mammalian target of rapamycin complex 1 (mTORC1) activity is elevated in preclinical and clinical models of TSC (123, 124). mTORC1 is classically known as a master regulator of mRNA translation to promote protein synthesis (123, 125); however, previous research denotes mTORC1 has equal responsibilities in promoting and repressing mRNA translation (126). Treatment with rapamycin, an mTORC1 inhibitor, caused an upregulation of 166 proteins. Termed mTOR-OFF proteins, this select population of mRNAs are translated when mTORC1 is in its inactive form. One of those mTOR-OFF proteins is KCC2 where the *SLC12A5* mRNA is translated when mTORC1 is repressed. Since mTORC1 activity is aberrant in TSC, it can be hypothesized that reduced KCC2 protein synthesis is under direct influence of dysregulated mTORC1 activation (Figure 4). However, previous evidence also indicates that rapamycin treatment represses the protein expression of KCC2 (127). Discrepancies between these two findings can be due to the dosage and frequency of rapamycin treatments. The first study (126) used a one-hour treatment of 10 mg/kg rapamycin prior to tissue collection while the second study (127) used a three-day regimen of 5 mg/kg rapamycin. A single treatment of rapamycin can be considered ‘acute,’ only inhibiting mTORC1 of the mTOR complex, while doses across multiple days can be considered ‘chronic’ inhibiting both mTORC1 and mTORC2 (128). Further experimentation to inhibit only mTORC2 activity will properly address the confounding results.

Interestingly, previous studies highlight a distinct relationship between NKCC1 and mTORC1 activity. NKCC1 activity represses mTORC1 through the Leu transporter LAT1-4F2hc (LAT1), as well as the Akt and Erk signaling pathways (129). In models of TSC where mTORC1 activity is upregulated, NKCC1 upregulation as denoted by previous research (118, 119) could serve as a compensatory mechanism to normalize mTORC1 activity to homeostatic levels. This study also denotes that NKCC1 activity positively correlates with cell size. Cellular hypertrophy is a recognized phenotype in models of TSC (130), however, this study indicates that elevated NKCC1 expression could contribute to increased soma size.

### Additional idiopathic forms of autism spectrum disorder

In conjunction with the previously discussed research, other idiopathic forms of ASD have been identified to display disturbances in GABAergic signaling. Preclinical models of Down Syndrome (Ts65Dn) and DiGeorge Syndrome (Lgdel^+/−^) exhibit a depolarizing GABA_A_ receptor mediated response or a delay to switch to hyperpolarizing GABAergic responses (131, 132). These electrophysiological findings are also correlated with an increased ratio between NKCC1 and KCC2 expression (131). Treatment with bumetanide in Ts65Dn mice furthermore restored inhibitory GABAergic responses while simultaneously ameliorated LTP deficits and cognitive dysfunction (133). While there is a strong argument supporting the presence and impacts of disturbed GABAergic signaling in ASD, further exploration in other idiopathic forms and preclinical models of ASD can corroborate a conserved mechanisms underlying depolarizing GABAergic responses across heterogenous ASD populations.

## Substance use disorders

Substance Use Disorders (SUD) are classically characterized by compulsive drug-seeking behaviors, loss of control to limit substances, as well as negative emotional behaviors during withdrawal states (134). The underlying neurocircuitry is executed through the mesolimbic dopaminergic reward pathway which involves projecting dopaminergic neurons from the ventral tegmental area (VTA) to the nucleus accumbens (NAc) (135). While dopamine is responsible for instigating the reinforcing properties of substances, local GABAergic interneurons that synapse onto dopaminergic neurons in the VTA tightly regulate the activity of the mesolimbic pathway (136, 137). Previous reports indicate several substances inhibit GABAergic interneuron activity, disinhibiting dopaminergic neurons to elevate dopamine levels (135, 138–140). However, prolonged exposure to substances results in a ‘hypodopaminergic state,’ promoting drug-seeking behaviors to normalize dopamine levels during withdrawal (141–144). Moreover, collective evidence suggests that substance-induced hypodopaminergia could result from sustained activation of VTA GABAergic interneurons via an excitatory GABA switch.

### Alcohol use disorder

Alcohol Use Disorder (AUD) is broadly characterized by repetitive consumption of ethanol with a heighted vulnerability to seek alcohol following the cessation of drinking (145, 146). The reinforcing properties of ethanol on the nervous system are highly controlled by dopaminergic signaling, as described previously (135). However, repetitive drinking behaviors and subsequent alterations in dopaminergic circuity in preclinical models are hypothesized to be driven by excitatory GABAergic signaling.

A previous case study (147) highlighted a 50-year-old male patient undergoing alcohol withdrawal. Treatment with 1 mg lorazepam, a GABA_A_R positive allosteric modulator, resulted in paradoxical behavioral outcomes that included anxiety, agitation, and eccentric behaviors. In conjunction to this human study, several contributions have been made in preclinical models to understand the molecular mechanisms underlying a paradoxical GABAergic effect following alcohol consumption. Previous research (29, 148) shows that alcohol promotes an excitatory switch amongst GABAergic neurons in the ventral tegmental area (VTA). Following both two-bottle choice paradigms and acute ethanol injections, mice displayed a positive shift in the GABA reversal potential as well as dysfunctions in chloride transport in VTA GABAergic neurons. Additional studies have pointed to reductions in KCC2 and pKCC2 expression in the VTA following ethanol exposure (149). Of note, ethanol exposed rodents treated with CLP290, a KCC2 activator, diminished further ethanol consumption, while consumption was enhanced following treatment with VU0240551, a KCC2 inhibitor. These results furthermore highlight the importance of KCC2 expression in maintaining inhibitory GABAergic responses and regulating the reinforcing properties of ethanol.

Imbalances in chloride homeostasis have also been reported in other brain regions following ethanol exposure. Studies have shown increases in NKCC1 expression in the hippocampus (150, 151). Disturbances in excitability and the presence of epileptiform activity have been reported in the limbic system following ethanol exposure (152), however, the previously discussed data hypothesizes an underlying molecular mechanism relating to chloride dyshomeostasis.

### Nicotine

Similar to preclinical studies following ethanol exposure, previous research has reported disturbances in GABAergic signaling following nicotine exposure. Reports indicate that nicotine treatment in rodents is followed by a depolarizing shift in GABAergic signaling in VTA GABAergic neurons (153, 154). The presence of excitatory GABAergic responses is supported with results showing a reduction in KCC2 expression within the VTA following nicotine exposure. Interestingly, cotreatment of nicotine and CLP290 restores hyperpolarizing GABAergic signaling and further diminishes self-administration of reinforcing substances. Chronic nicotinic exposure during neonatal development (P1–P5) was additionally shown to increase hippocampal KCC2 mRNA expression in only males, and not females (155), indicating a potential interaction of sex on the effects of nicotine on chloride homeostasis.

Although nicotine’s effect on promoting excitatory GABAergic signaling is primarily hypothesized to be linked to imbalances in chloride homeostasis, it is important to also consider the contributions of nicotinic acetylcholine receptors (nAChRs). In the immature brain, nAChRs aid in transitioning GABAergic signaling from excitatory to inhibitory. Specifically, previous research indicates α7-containing nAChRs (α7-nAChRs) are key players in terminating the depolarizing effects of GABA. In a α7KO preclinical model, the switch from depolarizing GABA is delayed with subsequent increases in NKCC1 and decreases in KCC2 expression in the mature brain. Of note, a loss of α7-nAChRs further results in defects in neuronal migration and maturation within both the central and peripheral nervous system (156, 157).

The α7-nAChRs are highly susceptible to desensitization following chronic exposure to nicotine (158). As previously noted (154), adolescent exposure to nicotine drastically influences neuronal function and drug-seeking behaviors into later stages of adulthood. Nicotine’s effects on these detrimental processes are mediated by disturbing chloride homeostasis during adolescence. Furthermore, it is important to consider the potential effects of nACR desensitization, following chronic nicotine exposure, as an alternative mechanism promoting excitatory GABAergic signaling in the reward neurocircuitry and the overall reinforcing properties of nicotine.

### Opiates

Opiates are classically considered a highly addictive substance and exert their effects through the mesolimbic dopaminergic reward pathway (135, 159). The reinforcing properties of opiates on the reward pathways are hypothesized to be linked to excitatory GABAergic signaling in the VTA. Previous studies report a population of VTA GABAergic neurons that depolarize upon GABA_A_R activation following opiate withdrawal (160–162). The depolarizing GABAergic effects of opiates are also paired with reduced inhibitory GABAergic tone (163), as well as deficits in chloride exportation (164). Moreover, several molecular mechanisms are hypothesized to promote an opiate-induced excitatory switch. One, opiate-dependent/withdrawn models display reduced KCC2 expression in VTA GABAergic neurons (163, 164), reducing extracellular chloride concentration upon GABA_A_R activation. Two, increased phosphorylation of cAMP Response Element-Binding Protein (CREB) in the VTA during opiate-dependence/withdrawal (160), can promote excitatory actions of GABA_A_Rs (165). Three, opiate exposure can elevate carbonic anhydrase activity (160), leading to an accumulation of intracellular bicarbonate ions. Greater intracellular bicarbonate ions are therefore exported from the neuron through activated GABA_A_R, further resulting in a depolarizing GABAergic response.

### Excitatory GABA_B_ receptor signaling and substance use disorders

**Figure 5:**
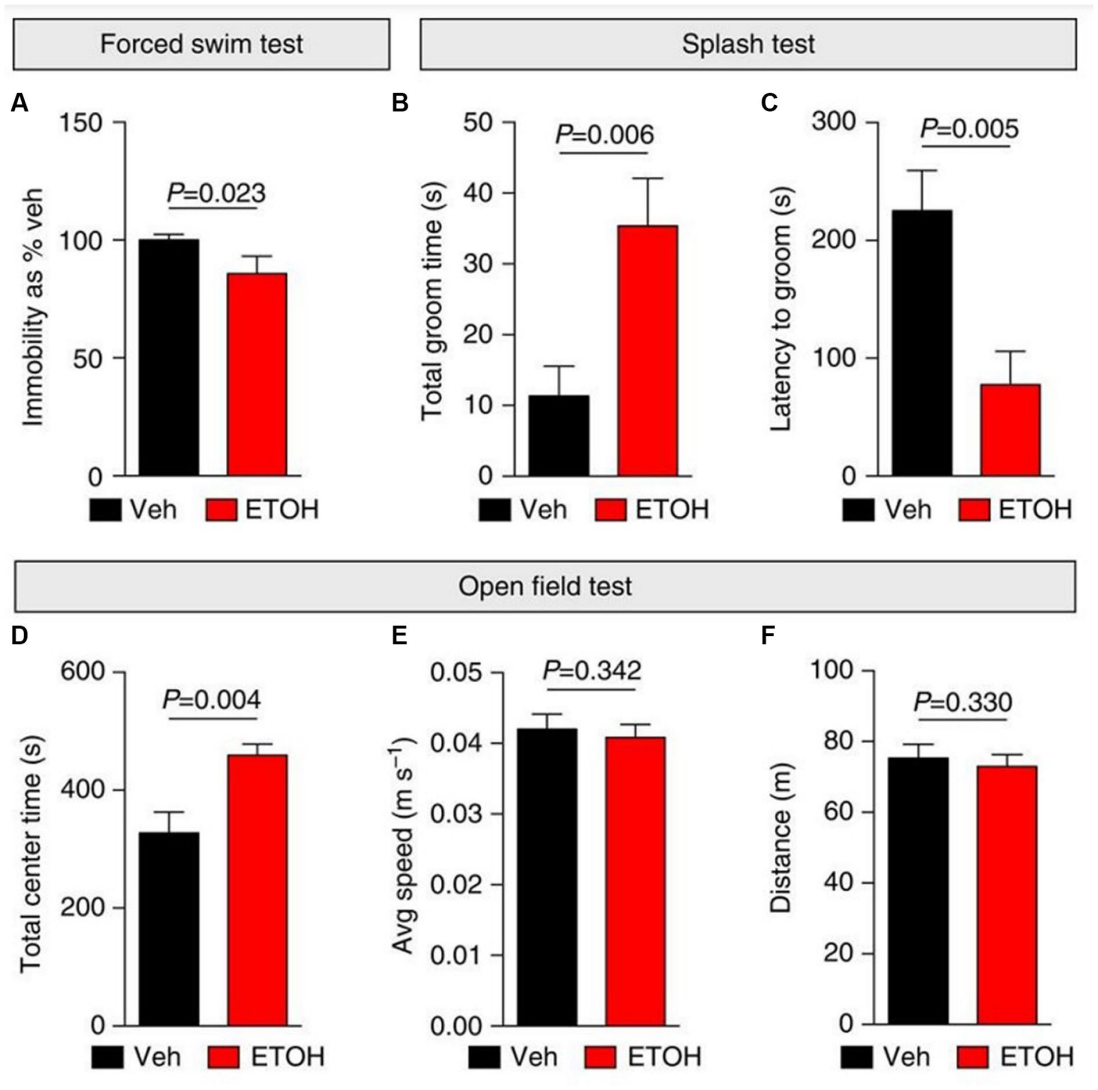
Ethanol elicits anti-depressant behavioral effects in mice. **(A–F)** Depressive-like (self-care and behavioral despair) and anxiety-like behaviors were measured 24 h following i.p. injections with vehicle (Veh; saline) or ethanol (ETOH; 2.5 g kg^−1^) in mice. **(A)** Ethanol elicited anti-depressant behavioral effects by decreasing immobility during a forced swim test (FST) and increasing self-grooming behaviors during a splash test **(B,C)**. **(D–F)** An open field test was used to measure anxiety-like behaviors following ethanol treatment. **(D)** Ethanol induced anxiolytic behaviors by increasing the time spent in the center of the open field. Additionally, ethanol did not affect mobility in mice **(E,F)**. Figure reused from (166).

**Figure 6:**
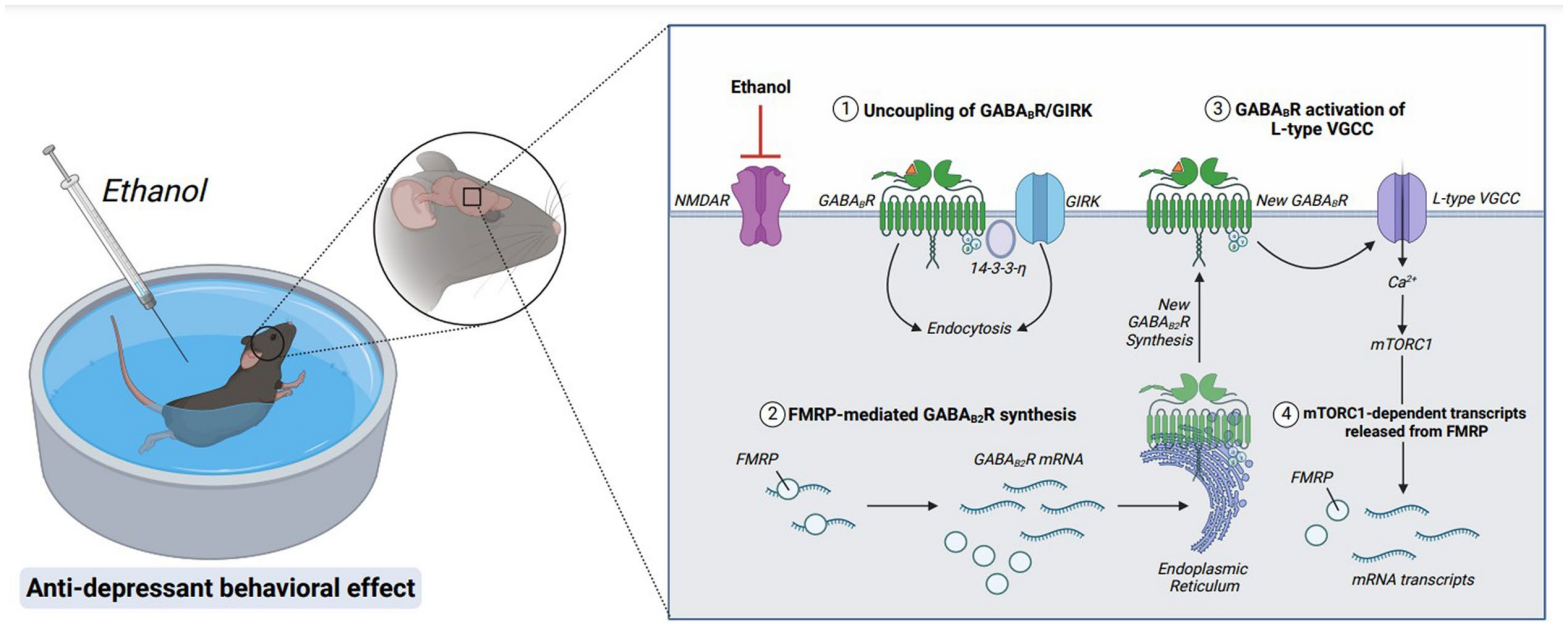
Molecular mechanisms underlying the rapid antidepressant properties of ethanol. Acute ethanol exposure antagonizes NMDAR signaling. GABA_B_Rs are then uncoupled from GIRK channels and endocytosed via stabilization of adaptor protein, 14-3-3-η. GABA_B2_R mRNA, a target of RNA-binding protein FMRP, is released from translational repression, and new GABA_B2_R protein is synthesized. A new population of surface GABA_B_Rs activate L-type voltage-gated calcium channels (L-type VGCC), depolarizing the membrane via calcium influx and activating mTORC1 signaling. Furthermore, mTORC1-dependent transcripts are released from FMRP suppression, upregulating transsynaptic proteins required for anti-depressant behavioral effects. Figure created with BioRender.com.

Acute exposure to ethanol has been previously shown to result in an anti-depressant behavioral effect (Figure 5) (166–168). Patients with AUD are hypothesized to continue drinking in an attempt to avoid negative affect states as the anti-depressive properties of ethanol shift to depressive properties during withdrawal or chronic use (134, 169). Ethanol has been shown to have very similar effects to rapid antidepressants as both antagonize excitatory NMDARs and affect GABAergic neurotransmission (166). Rapid antidepressants that are NMDAR antagonists stabilize GABA_B_ receptors (GABA_B_Rs) via an intracellular adaptor protein, 14-3-3η. Stabilization of 14-3-3η leads to the decoupling of GABA_B_Rs from inwardly rectifying potassium channels (Kir/GIRK), causing GABA_B_Rs to increase dendritic calcium through L- type voltage-gated calcium channels (170). Increasing dendritic calcium additionally activates the mammalian target of rapamycin (mTOR) kinase activity in dendrites. mTOR, which is a serine/threonine protein kinase, promotes the synthesis of plasticity-related proteins, which is required for the antidepressant efficacy of NMDAR antagonists (Figure 6) (170). Studies have demonstrated that blocking GABA_B_Rs prevent the effects of rapid anti-depressants, decrease the activity of mTOR, and reduce the synthesis of plasticity-related proteins such as brain-derived neurotrophic factor (BDNF) (171). Thus, an excitatory switch in GABA_B_Rs play a critical role in the efficacy of antidepressants by increasing protein synthesis, as well as increasing dendritic calcium signaling (166, 170).

These data suggest that ethanol exerts its rapid antidepressant effects through similar cellular mechanisms as NMDAR antagonists: promoting a depolarizing response from GABA_B_Rs. In addition to altering GABA_B_Rs to switch from opening inwardly rectifying potassium channels to increasing dendritic calcium, ethanol also promotes upregulation of GABA_B_Rs (166). Of note, the increased surface expression of GABA_B_Rs requires FMRP (Figure 6). Without FMRP, the ethanol-induced changes in GABA_B_R expression and excitatory signaling are absent, suggesting that FMRP is important in enhancing the antidepressant properties of ethanol (166).

Moreover, these studies provide a novel mechanism demonstrating an unprecedented role of excitatory GABA_B_R signaling towards the reinforcing properties of ethanol.

## Discussion

The data discussed in this review highlights the presence of paradoxical, excitatory GABAergic signaling as a potential contributor toward disease pathology in various neurological disorders. Although excitatory GABAergic responses are critical in the maturation of the nervous system, several disorders can arise due to the prolonged display or inability to terminate depolarizing GABAergic signaling. The predominant mechanism underlying an inability to transition from excitatory to inhibitory GABA across the discussed neurological disorders can be traced to imbalances in chloride ion homeostasis. Moreover, the common denominator contributing to chloride dyshomeostasis is the downregulation of KCC2 and an upregulation of NKCC1. Table 1 outlines potential mechanisms underlying excitatory GABAergic signaling across neurological disorders.

**Table 1:** Comparing mechanisms underlying excitatory GABAergic signaling in neurological disorders.

Model	Region	GABAergic physiology	Hypothesized mechanisms	References
*Stress-related disorders*				
Restraint stress	Hypothalamus	Depolarizing shift in E_GABA_ Increased GABA_A_R-mediated AP frequency	KCC2 downregulation Reduced KCC2 phosphorylation (Ser940) Neurosteroidgenesis (THDOC) Reduced GABA	(17, 27, 32, 50)
	Hippocampus	Depolarizing shift in E_GABA_	Reduced KCC2 phosphorylation (Ser940) Reduced GABA_A_R subunits	(30, 43)
	VTA	Depolarizing shift in E_GABA_ Increased GABA_A_R-mediated AP frequency	Reduced KCC2 phosphorylation (Ser940)	(29, 31)
Maternal separation	Hippocampus	Increase in GABA_A_R-mediated [Ca^2+^]_i_	NKCC1 upreguation KCC2 downregulation	(24, 37, 49)
Hypersomatic Stress	Hypothalamus	Depolarizing shift in E_GABA_	NKCC1 upregulation	(28)
CUMS	Hypothalamus	Depolarizing shift in E_GABA_	NKCC1 upregulation KCC2 downregulation Reduced GAD65 Increased GAT-1 Reduced GABA_A_R subunits	(25, 36, 42)
	Hippocampus	ND	Reduced vGAT Reduced GABA_A_R subunits	(39, 40)
Prenatal stress	Cortex	Increased GABA_A_R-mediated spontaneous activity	KCC2 downregulation	(23)
Early-life stress	Hypothalamus	Depolarizing shift in E_GABA_	ND	(26)
	Cortex	Early hyperpolarizing shift in GABA	NKCC1 downregulation	(52)
Cold stress	Hypothalamus	ND	Reduced [^3^H]GABA binding reduced GAD67	(34)
Social-defeat stress	NAc	ND	Reduced vGAT	(38)
Repeated swim stress	Hippocampus	ND	Reduced GABA_A_R subunits	(41)
*Alzheimer’s disease and dementia*				
Humans	Cortex	ND	NKCC1 upregulation KCC2 downregulation	(77)
Aβ treatment	Hippocampus	Depolarizing shift in E_GABA_	NKCC1 upregulation KCC2 downregulation	(71, 81)
APP/PS1	Hippocampus	Depolarizing shift in E_GABA_	NKCC1 upregulation KCC2 downregulation	(71, 79, 80)
AD11	Hippocampus	Increased GABA_A_R-mediated AP frequency Positive shift in EGPSCs	KCC2 downregulation	(76)
APOE4-KI	Hippocampus	ND	NKCC1 upregulation	(83)
*Autism Spectrum Disorders*				
Humans	Cortex	ND	Nonfunctional *SLC12A5* mRNA variants	(90)
Fragile X Syndrome (*Frm1* KO)	Cortex	Depolarizing shift in E_GABA_ Increase in GABA_A_R-mediated [Ca^2+^]_i_ fluorescence	NKCC1 upregulation Reduced KCC2 phosphorylation	(96–98, 101)
	Hippocampus	Depolarizing shift in E_GABA_ Increased GABA_A_R-mediated AP frequency	KCC2 downregulation	(99)
Rett Syndrome (*Mecp2* KO)	Hippocampus	Depolarizing shift in E_GABA_ Increased GABA_A_R-mediated AP frequency	KCC2 downregulation REST-mediated impairment of SLC12A5 mRNA translation	(99, 104–109)
Tuberous sclerosis complex (*Tsc1* KO)	Cortex	Increased GABA_A_R-mediated AP frequency Depolarizing shift in E_GABA_	NKCC1 upregulation KCC2 downregulation NKCC1/KCC2 dysregulation via aberrant mTORC1 activity	(118, 119, 126, 127, 129)
Down Syndrome (*Ts65Dn*)	Hippocampus	Increase in GABA_A_R-mediated [Ca^2+^]_i_ fluorescence Increased GABA_A_R-mediated spontaneous activity Increased GABA_A_R-mediated AP frequency Depolarizing shift in E_GABA_	NKCC1 upregulation	(132, 133)
DiGeorge Syndrome (*Lgdel^+/−^*)	Hippocampus	Increase in GABA_A_R-mediated activity	KCC2 downregulation	(131)
*Substance use disorder*				
Alcohol use disorder	VTA	Depolarizing shift in E_GABA_ Impaired chloride export	KCC2 downregulation Reduced KCC2 phosphorylation (S940)	(148, 149)
	Hippocampus	Depolarizing GABA_B_R signaling by activating L-type VGCC	NKCC1 upregulation	(150, 151, 166)
Nicotine	VTA	Depolarizing shift in E_GABA_ Increased GABAAR-mediated AP frequency	KCC2 downregulation α7-nAChR desensitization	(153, 154, 158)
	Hippocampus	ND	KCC2 upregulation in males, not females	(155)
Opiates	VTA	Increased GABAAR-mediated AP frequency Impaired chloride export	KCC2 downregulation Increased CREB phosphorylation Elevated carbonic anhydrase activity	(16, 160–163)

Summary of data outlining excitatory GABAergic signaling in stress-related disorders, Alzheimer’s disease, Autism Spectrum Disorders, and substance use disorders with hypothesized molecular mechanisms. “ND” indicates that excitatory GABA has not yet been determined in the respective model and region. *Abbreviations*: Ventral Tegmental Area (VTA), Nucleus Accumbens (NAc), Action Potential (AP), K+/Cl-cotranspoter 2 (KCC2), Tetrahydrodeoxycorticosterone (THDOC), Na+/K+/Cl-cotransporter 1 (NKCC1), Glutamic acid decarboxylase (GAD67/65), Gamma-aminobutyric acid transporter-1 (GAT-1), Vesicular GABA transporter (vGAT), Repressor element 1 silencing transcription factor (REST), Mammalian target of rapamycin complex 1 (mTORC1), α7-containing nicotinic acetylcholine receptors (α7-nAChR), cAMP Response Element Binding Protein (CREB).

Several disorders discussed have comorbid symptoms that could be caused by dysregulated KCC2 and NKCC1 expression. For example, stress is associated with anxiety and substance use disorders (172, 173), cognitive dysfunction is present in both stress-related disorders and AD (174, 175), and epileptogenesis occurs in both autism spectrum disorders (ASD) and Alzheimer’s disease patients (176–178). All these overlapping symptoms could be hypothesized to have common molecular mechanisms linked to KCC2/NKCC1 expression and chloride homeostasis. It is important to note that bumetanide, an NKCC1 inhibitor, has shown positive therapeutic effects in alleviating symptoms in ASD, Alzheimer’s disease, and stress-related disorders (49, 84, 93, 120–122). Furthermore, NKCC1 inhibition could serve as a universal target to prevent pathologies and symptom onset in clinical populations for various neurological disorders.

It is also critical to discuss that while chloride dyshomeostasis could be the predominant cause of excitatory GABAergic signaling in disorders, reductions in transsynaptic GABAergic signaling should also be considered a potential factor. While the data presented in this review outlined the contributions of downregulated GABA signaling towards stress-induced excitatory GABA_A_R-mediated responses, reductions in GABAergic signaling are also present in Alzheimer’s disease (179), ASD (100, 180), SUD (181, 182). It can be hypothesized that repurposing therapeutics that enhance endogenous levels of GABA could therefore alleviate excitatory GABA_A_R signaling throughout various disorders.

**Figure 7:**
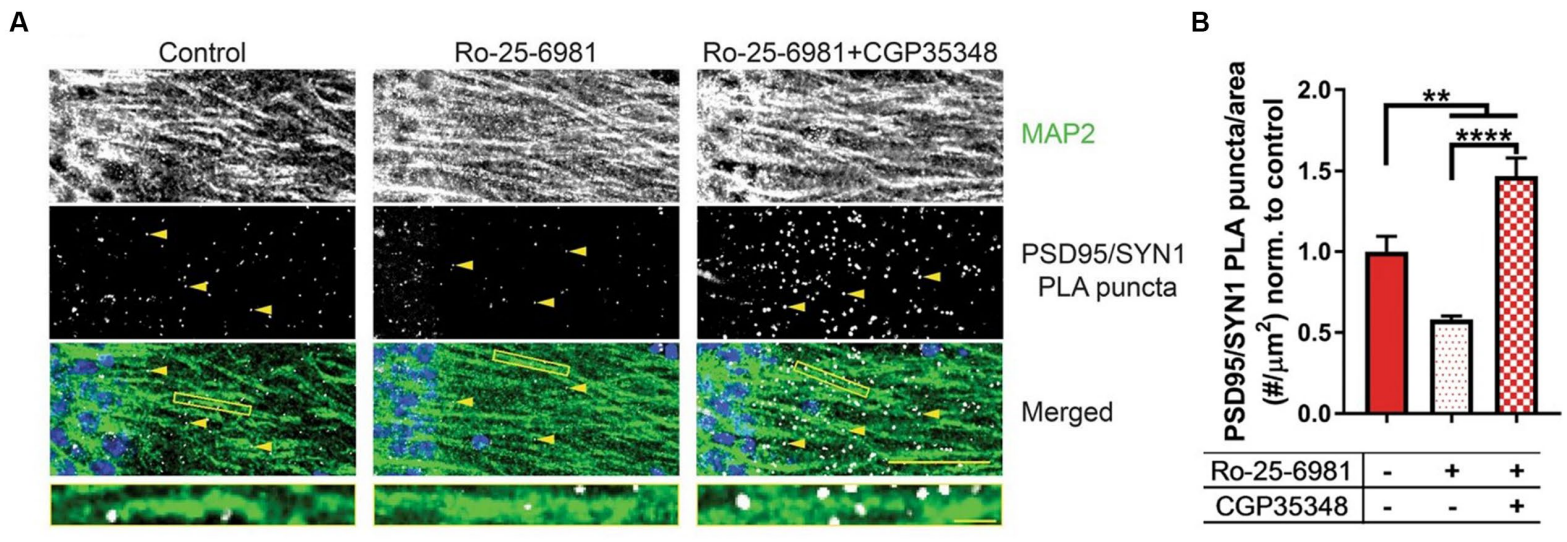
Combinational treatment between Ro-25-6981 and CGP35348 increases hippocampal synapse number in an *Fmr1* KO mouse. **(A)** Representative images of CA1 hippocampal sections from *Fmr1* KO mice. MAP 2, a marker of dendrites, is denoted in the first row, while synapses are denoted by the yellow arrows in the second row (PSD95/SYN1 PLA puncta). Synapses are detected in slice using a novel method, DetectSyn, which uses proximity ligation assay (PLA) technology and immunofluorescence to note the presence of synaptic engagement. Bottom row includes a merged image with MAP2 in green, PLA puncta (synapses) in white, and DAPI in blue. **(B)** Quantification of synapse number following treatments in *Fmr1* KO hippocampal slice. Compared to control (*red*), treatment with Ro-25-6981 (*light red*), NR2B antagonist, reduced the number of synapses in *Fmr1* KO slice. Treatment with Ro-25-6981 and CGP35348, GABA_B_R antagonist, increases synapse number as compared to control (*checkered red*). Figure reused from (183).

Although the studies included in this review focused on the excitatory actions of GABA via ionotropic GABA_A_Rs, there is a lack of understanding indicating whether metabotropic GABA_B_Rs also behave in a similar fashion in neurological disorders. As noted, the antidepressive properties of both ethanol and rapid antidepressants share similar molecular mechanisms in which GABA_B_Rs are uncoupled from GIRK channels to L-type voltage-gated calcium channels, resulting in depolarization. Ethanol and rapid antidepressants, moreover, require FMRP to switch GABA_B_Rs from inhibitory to excitatory (166, 183). Following exposure to ethanol, FMRP levels are additionally reduced in neuronal dendrites (166). It is important to also include that *SLC12A2* mRNA, the transcript encoding NKCC1, is a target of FMRP (95). It could therefore be hypothesized that ethanol-induced downregulation of FMRP could subsequently increase NKCC1 to cause a disturbance in chloride homeostasis, exacerbating the reinforcing properties for ethanol. Previous work (183) presents a novel therapeutic strategy to alleviate synapse loss in a model where FMRP is absent. A combination of both Ro-25-6981 (Ro), a GluN2B-specific antagonist, and CGP35348 (CGP), a GABA_B_R receptor antagonist, increased synapse number and rescued depressive-like symptoms in *Fmr1* KO mice (Figure 7). Changes in synapse number and behavior could theoretically be linked to the efficacy of Ro + CGP combinational therapies to rescue chloride ion imbalances via FMRP. Reduced FMRP levels have been shown in the neurological disorders discussed in this review (184–188), but also several other disorders not mentioned such as major depressive disorder (189), Parkinson’s disease (190), epilepsy (185, 191), and schizophrenia (192, 193). Furthermore, a novel combinational therapy antagonizing both NMDARs and GABA_B_Rs could universally alleviate pathologies and symptoms across various diseases.

The literature discussed presents an understanding towards the underlying molecular alterations resulting in excitatory GABAergic signaling in neurological disease states. Furthermore, the comorbid mechanisms affecting chloride homeostasis, GABA transsynaptic signaling, and GABAergic plasticity can prompt the repurposing of current FDA-approved therapeutics to alleviate symptom severity and pathogenesis within varieties of neurological disorders.

## Author contributions

CM: Writing – original draft, Writing – review & editing. AA: Conceptualization, Writing – original draft. CH: Conceptualization, Writing – original draft. KR-G: Conceptualization, Writing – review & editing.
